# Case of Hereditary Papillary Renal Cell Carcinoma Type I in a Patient With a Germline *MET* Mutation in Russia

**DOI:** 10.3389/fonc.2019.01566

**Published:** 2020-01-21

**Authors:** Dmitry S. Mikhaylenko, Alexey V. Klimov, Vsevolod B. Matveev, Svetlana I. Samoylova, Vladimir V. Strelnikov, Dmitry V. Zaletaev, Ludmila N. Lubchenko, Boris Y. Alekseev, Marina V. Nemtsova

**Affiliations:** ^1^Laboratory of Medical Genetics, Institute of Molecular Medicine, Scientific Biotechnological Park of Biomedicine, Sechenov University, Moscow, Russia; ^2^Laboratory of Pathology and Molecular Genetics, N. Lopatkin Institute of Urology and Interventional Radiology – Branch of the National Medical Research Center of Radiology, Moscow, Russia; ^3^Laboratory of Epigenetics, Research Centre for Medical Genetics Named After Academician N. P. Bochkov, Moscow, Russia; ^4^Department of Urology, Institute of Clinical Oncology, N. N. Blokhin National Medical Research Center of Oncology, Moscow, Russia

**Keywords:** kidney cancer, papillary renal cell carcinoma, hereditary cancer syndrome, *MET* gene, germline mutation

## Abstract

Hereditary papillary renal carcinoma (HPRC) is a rare autosomal dominant disease characterized by the development of multiple papillary type I renal cell carcinomas. This hereditary kidney cancer form is caused by activating mutations in *MET*. Descriptions of patients with HPRC are scarce in the world literature, and no cases have been described in open sources in Russia. Here, we describe a 28-year-old female Russian patient with 7 and 10 primary papillary renal cell carcinomas in the left and right kidneys, respectively. The patient did not have a family history of any of the known hereditary cancer syndromes. A comprehensive medical examination was performed in 2016 including computed tomography and pathomorphological analysis. The observed tumors were resected in a two-step surgical treatment. In February 2019, no sign of disease progression was detected in follow-up medical examination. Molecular genetic analysis revealed the germline heterozygous missense variant in *MET*: c.3328G>A (p.V1110I; CM990852). We have discussed the biological effects of the detected mutation and the utility of DNA diagnostics for treating patients with HPRC.

## Introduction

Renal cell carcinoma (RCC) shows the 9th highest incidence among all cancers worldwide and is a pressing problem in modern oncology (1). RCC due to germline mutations is a hereditary cancer syndrome accounting for only a few percent of all RCC cases, but has several unique features, both from the clinical (early manifestation, bilateral or multifocal tumors, and characteristic tumor type) and molecular genetics perspectives. Detection of a germline mutation in a proband is the main diagnostic test in the medical genetic counseling of patients with hereditary RCC. Thus, it is important to accumulate and systematize information on the relevant mutations and their phenotypic expression. Approximately 10 monogenic hereditary RCC forms have been described to date and can be diagnosed by direct DNA testing (2). Particularly, hereditary papillary renal carcinoma (HPRC, or PRCC1, OMIM 605074) is an autosomal dominant disease characterized by the development of multiple papillary type I renal cell carcinomas. This hereditary RCC form is caused by activating mutations in the *MET* proto-oncogene on chromosome 7q31 (3, 4). *MET* encodes for a receptor of the hepatocyte growth factor (HGF), which affects many cell types despite its name. *MET* mutations cause constitutive activation of the cytoplasmic domain of the receptor and stimulate cell division, which is considered as the main event in the carcinogenesis of papillary carcinomas in HPRC (5). Direct DNA diagnosis in HPRC is based on identifying mutations in *MET* exons 15–21, which code for the cytoplasmic domain of the receptor (6, 7). Studies of HPRC and germline *MET* mutations in Russian patients have not been described to date in the available literature. Here, we report the first clinical case of HPRC in Russia and its characteristics in terms of genetic diagnosis and treatment.

## Case Presentation

### Case History

A 28-year-old female patient (K.) was admitted to N. N. Blokhin National Medical Research Center of Oncology in June 2016 after being referred from another hospital for further diagnosis and being treated for multiple renal cell tumors. Patient K. gave informed consent to undergo diagnostic procedures and treatment, as well as to participate in the study, and for the presentation of relevant clinical and molecular data in this paper. This case report was approved by the local Ethics Committee at Sechenov University. Based on the medical records, the patient had pituitary adenoma with endo-, supra-, infra-, and latero-sellar growth with partial descending optic atrophy on the left in 2012. At that time, the disease was clinically manifested by broadening of the feet and fingers, increased sweating, cysts and diffuse changes in the thyroid gland, and an increase in the level of growth hormone. The pituitary adenoma was partly removed via endoscopic transsphenoidal surgery in 2012, and she was treated with somatostatin analogs. At the time of the follow-up examination in 2016, no pituitary adenoma recurrence was detected; she was recommended to continue taking the somatostatin analog (octreotide depot) 20 mg intramuscularly once every 28 days in combination with bromocriptine 2.5 mg per day. At the same time, multiple neoplasms were detected in both kidneys. Family history was negative. The patient and her immediate family had no oncological diseases at a young age or other signs suggesting any known cancer syndrome. At the time of the hospitalization of patient K. in the N. N. Blokhin National Medical Research Center of Oncology, her parents and the child did not have cancer symptoms.

### Instrumental Diagnosis

Patient K. was examined at Blokhin National Medical Research Center of Oncology. Computed tomography with intravenous contrast detected three 1–2 cm tumor lesions with the active accumulation of the contrast agent in the right kidney. In the left kidney, there were four tumor lesions: a 3.5 × 3.0-cm mostly cystic tumor with a soft-tissue component, with parietal accumulation of the contrast dye in the middle one-third portion; a tumor with a diameter of 1.3 cm at the upper pole; a tumor with a diameter of 1 cm in a subcapsular location in the middle one-third; and a tumor of 1.3 cm in diameter at the lower pole; these tumors similarly accumulated the contrast dye. Various tests were performed, including skeletal scintigraphy, computed tomography of thoracic organs, and ultrasound of the abdominal and pelvic organs, which showed no sign of distant tumor process. Blood count, chemistry, and clotting tests were carried out prior to surgery and showed no clinically significant abnormalities. Complex renal scintigraphy revealed an insignificant decrease in radionuclide clearance; preoperative creatinine clearance was 84 mL/min.

### Surgery

Based on the obtained diagnostic data, a multidisciplinary board was held, with the participation of surgical oncologist, oncologist, pathomorphologist, a specialist in radiation diagnostics and urologist. Taking into account the current recommendations of the European Association of Urology (8) and the current standards of medical care of the Ministry of Health of Russia for patient K. with bilateral localized kidney cancer, the tactics of stepwise tumor resection with preliminary examination using computed tomography with contrast was chosen.

Partial nephrectomy was performed in two steps. The right kidney was resected during the first step. Intraoperative findings included four tumors of 0.5–1.3 cm in dimension in the lower one-third, three tumors of 0.5–1.0 cm in the middle one-third, and three tumors of up to 1.5 cm in the upper one-third of the right kidney. Through hilar occlusion, all detectable tumors were consecutively removed with a margin of visually unchanged tissue and adjacent pararenal fat under intraoperative ultrasound guidance. The sides of each resection site were sutured together. The kidney ischemia time was 28 min, total surgery duration was 135 min, and blood loss was 300 mL. Postoperative complications did not occur; serum creatinine increased insignificantly to 118 μmol/L. Patient K. was discharged in satisfactory condition on day 13 after surgery.

**Figure 1:**
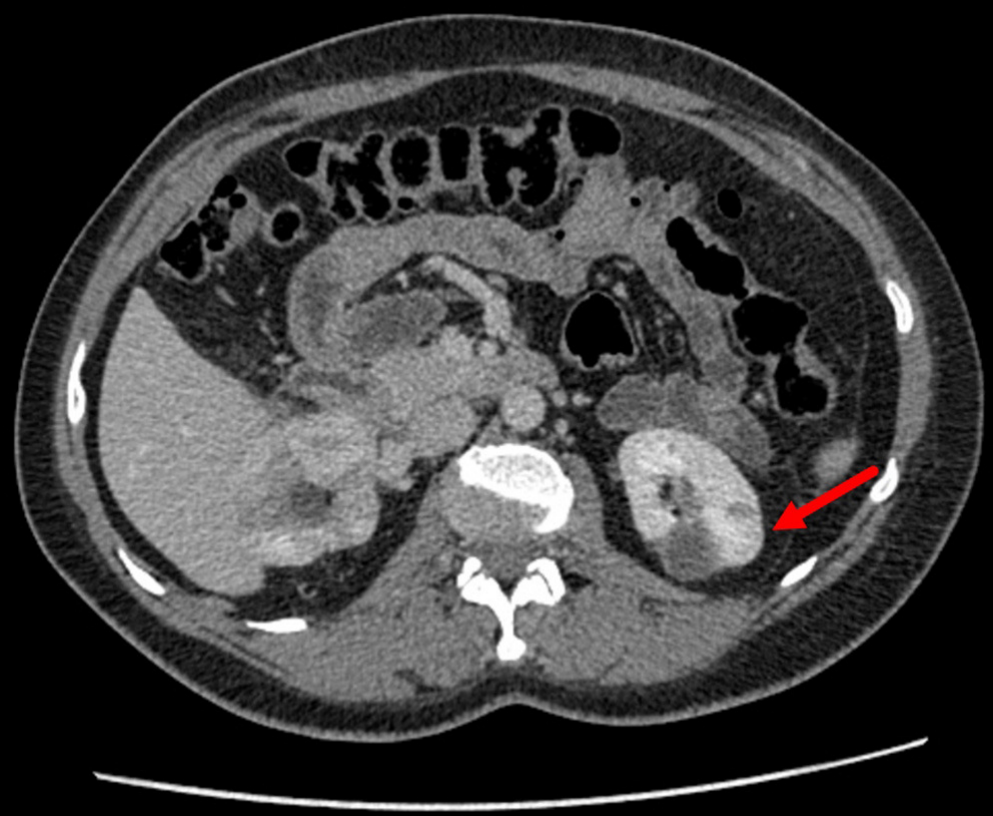
Contrast-enhanced CT of the kidneys in patient K. The left kidney examined prior to the second surgery is indicated.

Two months later, in August 2016, patient K. was again admitted to Blokhin National Medical Research Center of Oncology to undergo a second surgery. Computed tomography scanning of the abdomen with intravenous contrast was performed before surgery and showed a deformed right kidney with two low-density sites that had uneven outlines and were 3.0 × 2.2 cm in total (postoperative changes). At least seven contrast-accumulating nodular masses were detected in the left kidney. Compared to the previous scan, which was performed 2 months earlier, one large mass had increased to 4.0 × 3.2 cm (previously 3.7 × 3.0 cm) at the posterior surface, whereas the other masses remained unchanged (Figure 1). Complex testing showed no findings suggesting metastasis. In surgery, nine tumors were consecutively removed with a margin of visually normal tissue from the left kidney with hilar clamping (ischemia time 28 min). Blood loss was 300 mL, and surgery duration was 150 min. The patient developed a fever (38°C) 9 days after the surgery. Due to the dilation in the collecting system of the right kidney, a JJ ureteric stent was placed. The inflammatory infection worsened, warranting a change in the antibacterial therapy and a nephrostomy tube was placed on the right. Signs of acute pyelonephritis were eliminated. The nephrostomy tube was removed 18 days after the surgery. By the end of the surgical treatment, the creatinine clearance was 32 mL/min, and serum creatinine was 168 μmol/L. Patient K. was discharged in a satisfactory condition. Regular follow-up tests were performed in subsequent years. In February 2019, no sign of relapse or disease progression was detected in control complex testing, which included magnetic resonance imaging of the abdominal organs.

### Pathomorphological Diagnosis

**Figure 2:**
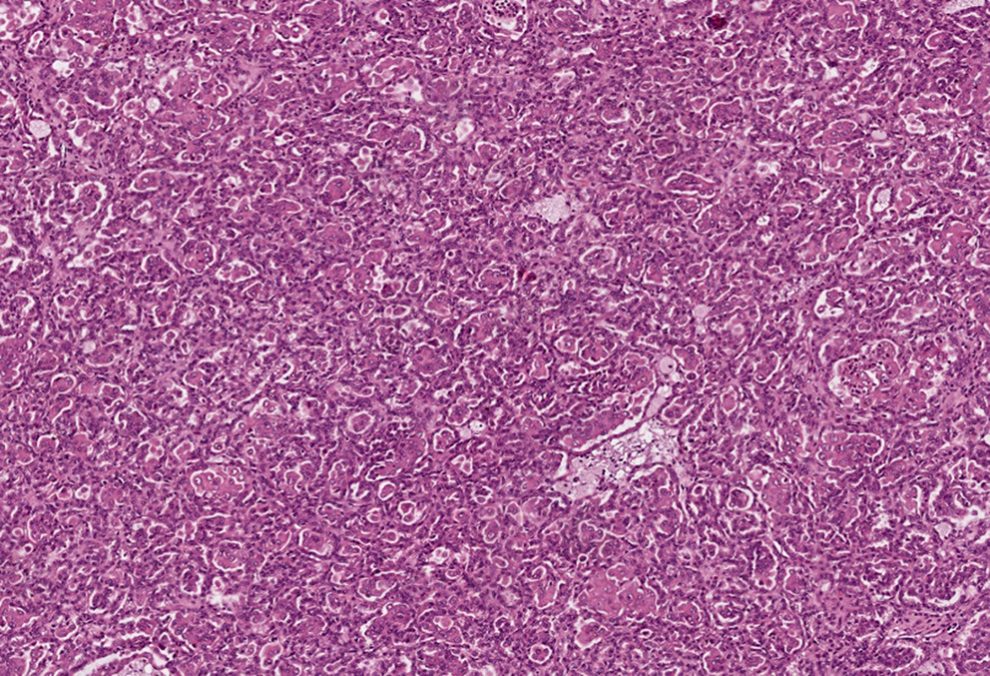
Pathomorphological examination of the mass excised from the right kidney of patient K. Type I papillary RCC. Hematoxylin–eosin staining, magnification ×100.

All tumor masses were removed from the right kidney during the first surgery. Microscopic examination revealed that they were structurally similar, and the tumors were identified as type I papillary RCC, Fuhrman grade 2 (Figure 2). Tumor cell growth was not detected in the pararenal fat. The nine tumors removed from the left kidney during the second surgery were also structurally consistent with type I papillary RCC, Fuhrman grade 2 by histology.

### Molecular Genetic Diagnosis

Because multiple type I papillary RCC tumors developed synchronously and bilaterally and affected a young patient, molecular genetic testing was carried out for patient K. in the period between the first and second surgeries. Note that it would be more advisable to do this before the first stage of the surgical intervention, so that in case of a positive test result, there would be an extra argument in favor of organ-preserving treatment, but the organizational peculiarities of the hospital care and the time frame for molecular genetic diagnostics made it possible to conduct a genetic study only during the first stage of treatment.

Genomic DNA was isolated from a peripheral blood sample with a DNA-sorb-B kit (NextBio, Moscow, Russia). Fragments of *MET* exons 15–21 were PCR-amplified using primers with previously published sequences (9). The reaction mixture contained 50–100 ng of genomic DNA, 2.5 mM MgCl_2_, 1.5 mM each dNTP, 2 pmol of the forward and reverse primers, 1 unit of thermostable Taq polymerase, and 5 μL of 5x PCR buffer (Interlabservice, Moscow, Russia); the total volume was 25 μL. Amplification was performed as follows: 95°C for 1 min; 35 cycles at 95°C for 45 s, 61°C for 25 s, and 72°C for 30 s; and final elongation at 72°C for 50 s. After amplification, the PCR product was treated with 1 unit of alkaline phosphatase and 4 units of *Escherichia coli* exonuclease I to eliminate free primers and dNTPs. The product was then subjected to Sanger sequencing using a BigDye Terminator v. 3.1 Cycle Sequencing kit and 24-capillary 3500xl sequencer (Thermo Fisher Scientific, Waltham, MA, USA).

**Figure 3:**
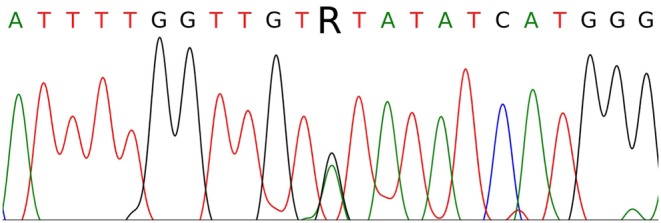
Sanger sequencing of part of *MET* exon 16 in patient K. The c.3328G>A (p.V1110I) mutation is indicated by the letter R.

The single-nucleotide substitution c.3328G>A was found in exon 16 in a heterozygous state (Figure 3). This substitution causes the missense mutation p.V1110I (Human Genome Mutation Database accession no. CM990852, http://www.hgmd.cf.ac.uk/ac/index.php). The germline mutation has been described as a cause of HPRC, occurs in the ATP-binding site of the HGF receptor, leads to its activation, and exerts a transforming effect in fibroblast cultures *in vitro* (10). The somatic p.V1110I mutation has similarly been found in a sporadic type 1 papillary RCC (Catalog of Somatic Mutations in Cancer accession no. COSM3724572, http://cancer.sanger.ac.uk/cosmic) and is considered as a pathological factor associated with activation of the HGF receptor (11, 12). These results confirmed the diagnosis of type I HPRC (OMIM 605074) in patient K. Following the results of the analysis, genetic counseling was carried out and mutation carriage testing was recommended for family members of the first line of kinship. Patient K. was also advised to consult a geneticist and a urologist once every 6–8 months. At the time of writing, there is no information about a confirmed mutation in any of the relatives of patient K. Together with a negative family cancer history, this suggests that the identified germline mutation can be classified as *de novo*.

## Discussion

The timing and extent of surgery in hereditary RCC are important as the timely excision of the tumor is essential considering that the patients are at a high risk of developing tumors in the same or contralateral kidney, which might then require repeated surgery and cause chronic kidney failure at a relatively young age. For example, up to 600 cysts and neoplastic growth microfoci may be found in the kidney of a patient with von Hippel Lindau syndrome (hereditary clear cell renal carcinoma). Currently, the standard method of treating patients with von Hippel Lindau syndrome confirmed by molecular genetic testing is removing the primary tumor via nephrectomy as soon as the tumor reaches 3 cm in the largest dimension, with certain contraindications (6, 13). However, early metastasis is possible in other hereditary RCC forms, warranting surgery immediately after diagnosis. For example, type II papillary RCC in hereditary leiomyomatosis and RCC (HLRCC, OMIM 150800B) often develop as a solitary unilateral tumor but are characterized by rapid progression and early metastasis; thus, the surgical treatment strategy in this disease is the same as in sporadic RCC. HLRCC is associated with mutations in *FH*, which is on chromosome 1q42 and codes for fumarate hydratase, a Krebs cycle enzyme. The same strategy is possible in type I HPRC because of the germline *MET* mutations. However, type I HPRCs are less malignant than type II tumors, enabling physicians to choose the proper surgical strategy (3, 14).

Type 1 multiple papillary carcinomas developed in the patient's kidneys at the age of 28. This is consistent with previous publications describing multiple lesions and early manifestation of HPRC as hereditary cancer, compared with a later age of manifestation of solitary unilateral sporadic papillary kidney carcinomas (2, 4, 6, 7). Apart from the differences in age of manifestation, HPRCs and sporadic papillary carcinomas of the kidney present several significant differences at the molecular genetic level. Even though *MET* germline mutations are the cause of type I HPRC, similar somatic *MET* mutations are found in sporadic tumors in no more than 20% of cases. At the same time, amplification of the 7q31 locus harboring *MET* occurs in 45%, and MET overexpression occurs in 90% of type I sporadic papillary carcinomas of kidney, which indicates its significant role in carcinogenesis of both hereditary and sporadic kidney tumors of this type (11). To date, the results of several large-scale studies of sporadic papillary renal cancer involving NGS methods have been published. In one of them, *MET* mutations were detected in 17% of type I tumors, and of the 17 detected mutations, 3 were not somatic but germline, which once again indicates the advisability of diagnosing HPRC in young patients with papillary RP even without a clearly traceable family history of the disease. As with clear cell renal cell carcinoma, papillary renal cell carcinoma in these studies demonstrated high mutation frequency in chromatin remodeling genes in both type I and type II tumors. Mutations in *SMARCB1, SETD2, ARID2, PBRM1*, and some other genes involved in the formation of SWI complex, and other chromatin modifiers were found in papillary RP with a frequency of 10–38% of cases. It is possible that mutations in genes that affect chromatin state increase genome instability and can be considered as driver mutations that act in the initial stages of carcinogenesis of type 1 papillary renal cell carcinoma (15–17).

Patient K. had a history of pituitary adenoma and acromegaly. Significant extrarenal clinical signs of the syndrome have not been observed in patients with HPRC according to recent reviews, in contrast to von Hippel Lindau syndrome, Birt–Hogg–Dube syndrome, and HLRCC (6, 13). It remains unclear whether the two conditions were associated with *MET* mutation in patient K.

Because *MET* activation is a key event in the pathogenesis of HPRC, its therapeutic potential has been suggested for targeted therapy with MET inhibitors in metastatic HPRC. Clinical studies of the various disease phases are currently underway to evaluate several targeted drugs, such as the synthetic MET inhibitors foretinib (XL880), tivantinib (ARQ197), and volitinib (HMPL-504), and anti-HGF monoclonal antibody rilotumumab (AMG-102). Studies of papillary RCC will be carried out with cabozantinib (XL184) and the ALK/MET/ROS/RET inhibitor crizotinib (5, 18). Promising results have been reported for foretinib as a targeted therapy for treating metastatic HPRC in ten patients; treatment efficacy was comparable or even higher than that in sporadic type I papillary RCC. The specific details of *MET* mutation in the ATP-binding site are an important consideration because subclass 1 inhibitors, such as crizotinib, do not affect certain mutant variants of the receptor, particularly the Y1230H variant (6, 19). The targeted MET inhibitor savolitinib led to a 4-fold increase in relapse-free survival in papillary RCC with *MET* mutation (20). Multikinase inhibitors (sunitinib, sorafenib, and axitinib) have been observed to induce more objective responses when used as targeted therapies to treat various non-clear cell RCCs, including papillary RCC, in the multicenter clinical study setting, and their combinations with MET or mTOR kinase inhibitors have been evaluated in clinical studies (21, 22). MET inhibitors may be applicable in targeted therapy of HPRC; especially foretinib, which has been shown to be effective in papillary carcinomas of the kidney harboring germline *MET* mutations (23).

## Conclusions

Diagnosis, including molecular genetic testing, and treatment of HPRC were performed in a Russian patient for the first time. The heterozygous germline p.V1110I activating mutation was identified in *MET* exon 16 in the patient. A positive result in molecular genetic testing for germline *MET* mutations may be considered as a weighty argument in favor of the choice of nephron-sparing surgery, in case genetic testing is performed prior to surgical treatment. In addition, detection of a germline mutation is necessary for a diagnosis of HPRC as it helps to provide further advice on regular examinations, allows for simpler testing of the mutation in the patient's close relatives, and may be important for planning targeted therapy with those with the mutation. Such testing can be advised to all young patients with multiple papillary carcinomas of the kidneys, even in the absence of a previous family cancer history and/or known frequencies of *MET* mutations in the population in question.

## Data Availability Statement

The datasets generated for this study can be found in the web portal of the Laboratory of Epigenetics, Research Centre for Medical Genetics at http://www.epigenetic.ru/projects/renal-cancer/16r-1190_D07_10.ab1.

## Ethics Statement

The studies involving human participants were reviewed and approved by the Local Ethics Committee at Sechenov University. The patients/participants provided their written informed consent to participate in this study.

## Author Contributions

DM performed molecular genetic diagnosis of patient K. and wrote abstract, introduction, and part of the case description in this manuscript. AK performed instrumental diagnosis, surgical treatment of patient K. and wrote clinical part of the case description. VM performed surgical treatment of patient K. SS provided pathomorphological examination of the removed tumors. VS prepared the illustrations. DZ wrote discussion in this manuscript. LL performed genetic counseling of patient K. BA supervised the clinical part of case study. MN wrote conclusion and supervised the laboratory part of case study.

### Conflict of Interest

The authors declare that the research was conducted in the absence of any commercial or financial relationships that could be construed as a potential conflict of interest.
